# Hypermethylation of *FOXP3* Promoter and Premature Aging of the Immune System in Female Patients with Panic Disorder?

**DOI:** 10.1371/journal.pone.0157930

**Published:** 2016-06-30

**Authors:** Martina Prelog, Deborah Hilligardt, Christian A. Schmidt, Grzegorz K. Przybylski, Johannes Leierer, Giovanni Almanzar, Nady El Hajj, Klaus-Peter Lesch, Volker Arolt, Peter Zwanzger, Thomas Haaf, Katharina Domschke

**Affiliations:** 1 Department of Pediatrics, University Hospital Wuerzburg, Wuerzburg, Germany; 2 Clinic for Internal Medicine C, University of Greifswald, Greifswald, Germany; 3 Institute of Human Genetics, Polish Academy of Sciences, Poznan, Poland; 4 Department of Internal Medicine, Medical University Innsbruck, Innsbruck, Austria; 5 Institute of Human Genetics, University of Wuerzburg, Wuerzburg, Germany; 6 Molecular Psychiatry, Department of Psychiatry, Psychosomatics and Psychotherapy, University of Wuerzburg, Wuerzburg, Germany; 7 Department of Psychiatry and Psychotherapy, University of Muenster, Muenster, Germany; 8 Department of Psychiatry and Psychotherapy, Ludwig-Maximilians-Universtät Munich, Munich, Germany; 9 kbo-Inn-Salzach-Klinikum, Wasserburg am Inn, Germany; 10 Department of Psychiatry, Psychosomatics and Psychotherapy, University Hospital Wuerzburg, Wuerzburg, Germany; CEA—Institut de Genomique, FRANCE

## Abstract

Immunological abnormalities associated with pathological conditions, such as higher infection rates, inflammatory diseases, cancer or cardiovascular events are common in patients with panic disorder. In the present study, T cell receptor excision circles (TRECs), Forkhead-Box-Protein P3 gene (*FOXP3*) methylation of regulatory T cells (Tregs) and relative telomere lengths (RTLs) were investigated in a total and subsamples of 131 patients with panic disorder as compared to 131 age- and sex-matched healthy controls in order to test for a potential dysfunction and premature aging of the immune system in anxiety disorders. Significantly lower TRECs (p = 0.004) as well as significant hypermethylation of the *FOXP3* promoter region (p = 0.005) were observed in female (but not in male) patients with panic disorder as compared to healthy controls. No difference in relative telomere length was discerned between patients and controls, but significantly shorter telomeres in females, smokers and older persons within the patient group. The presently observed reduced TRECs in panic disorder patients and *FOXP3* hypermethylation in female patients with panic disorder potentially reflect impaired thymus and immunosuppressive Treg function, which might partly account for the known increased morbidity and mortality of anxiety disorders conferred by e.g. cancer and cardiovascular disorders.

## Introduction

Anxiety disorders are among the most common mental health disorders in Europe in 2010 and confer a high individual and socioeconomic burden [1]. Anxiety disorders are chronic diseases ranking fifth regarding Years Lived with Disability (YLDs) among the 30 leading diseases and injuries in the United States in 2010 [2]. As a potential consequence of chronic stress, anxiety disorders have been shown to carry a high “allostatic load” [3], i.e. exert a physiological strain on organs and cells particularly pertaining to the cardiovascular system: For instance, phobic anxiety and increased anxiety levels, respectively, were found to be associated with an increased risk of coronary heart disease and cardiovascular death particularly in women [4–7]. This increased morbidity and mortality has in part been attributed to oxidative stress and inflammatory processes in anxiety disorders: Tension-anxiety symptoms were reported to correlate with an oxidative DNA damage marker [8], state/trait anxiety was associated with elevated C-reactive protein (CRP), interleukin-6 and fibrinogen levels [9], phobic anxiety in female patients with diabetes mellitus correlated with elevated inflammatory markers [10], patients with panic disorder showed significantly elevated peripheral proinflammatory cytokine and chemokine levels [11], and elevated inflammation as reflected by increased CRP levels was discerned to be associated with current anxiety disorders, particularly in male patients and patients with late-onset anxiety disorder [12].

Premature immunosenescence and a diminished regulatory T cell (Treg) function are discussed as etiopathological factors driving the immune system towards inflammatory diseases [13–17]: Aging of the immune system or ‘immunosenescence’ is characterized by loss of thymic function with decreased output of recent thymic emigrants (RTE) and increased replication of peripheral lymphocytes to compensate for the decrease in naive T cells. Elderly people are at risk for age-associated diseases, such as atherosclerosis and cardiovascular events, infectious diseases, cancer and inflammatory diseases due to breakdown of immune tolerance and higher inflammatory capacity. To estimate thymic function, T cell receptor excision circles (TRECs) have been shown to be useful markers due to their abundance in RTE and their proportional decline with age [18]. The number of naive T cells is maintained by peripheral proliferation of naive T cells which results in a dilution of TRECs [19,20].

Measurement of telomere lengths helps to estimate the individual replication history of cells. Telomeres are protective caps at the end of chromosomes and shorten with each cell cycle [21]. Indirectly, telomere shortening reflects the age of the singular immune cell and has been associated with susceptibility to age-related diseases, inflammation and also accelerated aging in mental disorders [22–24]. In detail, lower relative telomere length (RTL) has furthermore been reported to be associated with phobic anxiety in women and—with trendwise significance—also with items of the Crown-Crisp Index (CCI) mapping to panic and agoraphobia [25], with anxiety disorders particularly in older patients [26], and with anxiety disorders including generalized anxiety disorder, social phobia, agoraphobia and panic disorder after a two-year follow-up [27].

**Fig 1:**
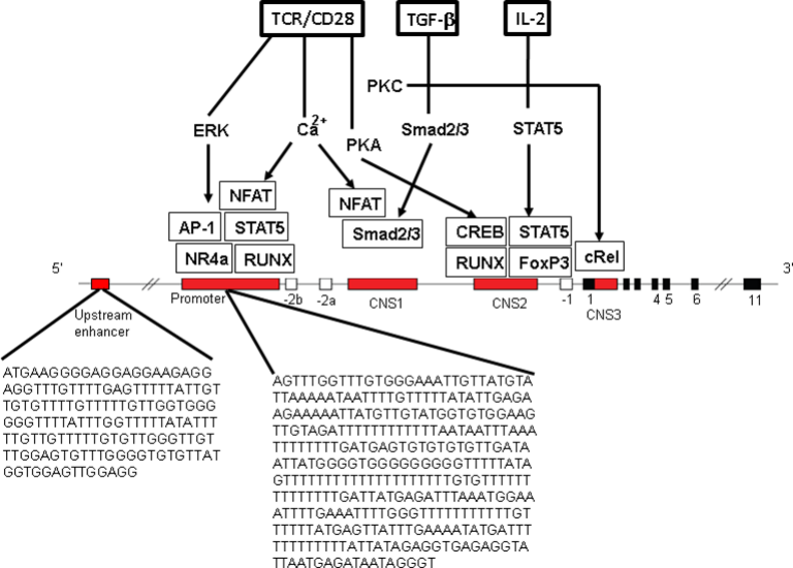
Molecules involved in *FOXP3* induction and stable expression and sequences analyzed. Enhancer (5’ upstream enhancer, conserved non-coding sequences, CNS1, CNS2 and CNS3 serving as enhancer regions) and promoter region are shown (modified after 36,37,68]. Promoter and enhancer regions are bound by several transcription factors and signals. A restriction in differentiation of nTregs is caused by protein inhibitor of the activated signal transducer and activator of transcription *STAT1* which binds to the *FOXP3* promoter and recruits DNA methyltransferase [68]. CNS1, an intronic enhancer (enhancer 1) is responsive to Tumor-growth-factor-beta (TGFβ) by Smad2/3 binding sites, close to the NFAT site, essential for differentiation of induced iTregs. CNS2, the T cell receptor (TCR)-responsive enhancer (enhancer 2) contains CpG islands and binding sites for transcription factors, *CREB* and *STAT5*. The unstable iTreg phenotype is associated with high methylation of the CNS2 region of Treg-specific demethylated regions (TSDRs). TSDR is a key factor in stability of Tregs [36]. Analyzed CpG regions were located in the 5’ upstream enhancer and the promoter of the *FOXP3* gene. Analyzed sequences are shown. The analyzed CpGs are located in the promoter region of *FOXP3* which do not contain any CpG island. The *FOXP3* enhancer lies in a CpG island. Abbreviations: EKR: extracellular signal regulated kinase, PKA: phosphokinase A, NFAT: Nuclear factor activated T cells, NR4a: Orphan nuclear receptor, RUNX: Runt-related transcription factor, CREB: CAMP responsible element binding protein 1, cRel: Proto-oncogene C-Rel, IL-2: interleukin-2, TCR: T cell receptor.

In inflammatory conditions, e. g. rheumatoid arthritis, signs of a prematurely aged immune system, e. g. lower TRECs in naive T cells and shorter telomeres in total lymphocytes, go ahead with quantitative and qualitative alterations of regulatory T cells [28,29]. The Forkhead-Box-Protein P3 (FoxP3) transcription factor is specifically expressed by naturally occurring regulatory CD25+CD4+ T cells (nTregs) and contributes to the immunosuppressive function of Tregs. Transiently FoxP3-expressing activated T cells (induced Tregs, iTregs) may be distinguished from nTregs by their methylation profile at the *FOXP3* promoter and enhancer regions [28,30]. Low *FOXP3* promoter methylation has been shown to be associated with highly CD25-expressing CD4+ Tregs [31]. Demethylated or hypomethylated CpG regions in promoter [31,32], upstream enhancer [33] or intronic enhancer [34] provides stable long-term expression of the *FOXP3* gene and, thus, is proposed to induce a stable Treg phenotype essential for maintaining Treg function to inhibit inappropriate or excessive immune responses [35]. Several molecules are involved in genetic and epigenetic regulation of *FOXP3* promoter and enhancer function (Fig 1) [35–37]. Along these lines, increased *FOXP3* methylation, resulting in decreased Treg levels, was observed in peripheral blood mononuclear cells (PBMCs) of patients with coronary artery disease [38]. Anti-CD25 antibody mediated depletion of Treg cells in mice has been shown to result in anxiety-like behavior in the elevated plus maze test and in higher serum IL-6 and TNF-alpha concentrations particularly after stress [39]. Development of inflammatory Th cell responses, a shift towards Th17 and reduced control by Tregs have been shown in individuals with generalized anxiety disorders [40–42].

In the present study, T cell receptor excision circles (TRECs), *FOXP3* methylation and relative telomere lengths (RTLs) were for the first time concurrently investigated in patients with panic disorder as compared to matched healthy controls in order to test for potential dysfunction and premature aging of the immune system in anxiety disorders. It was hypothesized that panic disorder would be associated with impaired thymus function as reflected by reduced TRECs as well as by *FOXP3* hypermethylation resulting in reduced immunosuppressive Treg function and accompanied by lower relative telomere length.

## Materials and Methods

### Samples

One hundred and thirty-one patients with panic disorder and 131 healthy, age- and sex-matched controls, recruited at the Department of Psychiatry and Psychotherapy, University of Muenster, Germany, were included into the study (Table 1). Diagnosis of panic disorder, in all cases the primary diagnosis at the time of inclusion, was ascertained by experienced psychiatrists on the basis of medical records and structured clinical interviews (SCID-I) according to the criteria of DSM-IV [1]. Individuals with mental retardation, neurological or neurodegenerative disorders impairing psychiatric evaluation as well as with severe somatic disorders were not included in this analysis. Medication with antidepressants and comorbidity with depression was recorded. Both cases and controls were of Caucasian ethnicity. Smokers were defined by the consumption of more than two cigarettes/day.

**Table 1:** Demographics.

	Patients		Healthy controls	
	Female (n = 85)	Male (n = 44)	Female (n = 85)	Male (n = 44)
**Age (years)**^**1**^	36.9 ± 10.8	34.1 ± 11.7	36.8 ± 10.9	34.1 ± 10.8
**Age at disease onset (years)**^**1**^	28.9 ± 11.3	28.2 ± 9.8	—	—
**Disease duration (years)**^**1**^^,^ ^**2**^	6.6 ± 6.6	6.5 ± 6.9	—	—
**Depression (yes/no/not documented)**	27/40/38	19/19/6	0/85/0	0/44/0
**Antidepressants (yes/no/not documented)**^**3**^	33/49/3	19/18/7	0/85/0	0/44/0
**Smoker status (yes/no/not documented)**	25/56/4	14/22/8	Not documented	Not documented

^1^ Values are given in mean ± standard deviation.

^2^ Disease duration significantly correlated with age (R = 0.434; p = 0.007) in male patients and near to significance in female patients (R = 0.224; p = 0.056).

^3^ Antidepressants: SSRIs: N = 46, Tricyclic Antidepressants: N = 2, NaSSA: N = 1, Melatonergic: N = 1, SSRI plus antipsychotics (off-label) N = 2.

The study was approved of by the ethics committee of the University of Muenster, Germany, written informed consent was obtained from all participating subjects, and the study was conducted according to the ethical principles of the Helsinki Declaration.

### Quantification of TRECs and relative telomere length

DNA was extracted from separated whole EDTA blood using the FlexiGene DNA Kit (QIAGEN, Hilden, Germany) according to the manufacturer's instructions. Signal-joint TREC concentrations were determined by PCR as described in detail previously [43,44]. Recombination-activating gene 2 (RAG2) was used as a reference gene to normalize the quantity of DNA used for real-time quantitative polymerase chain reaction (RQ-PCR).

Determination of relative telomere length (RTL) was performed by calculating the ratio of a quantitative PCR reaction product from the same sample using specific primers for telomeres and a single copy gene as described previously [45–47].

### Bisulfite pyrosequencing

Assays quantifying the methylation levels of CpGs in the target regions, i.e. *FOXP3* promoter (human build hg 19 Chromosome X, 49121152–49121485 bp, len: 333) and *FOXP3* 5’ upstream enhancer (CpG human build hg 19 Chromosome X, 49126597–49126750 bp, len: 153) (both ensemble releaser 15 February 2014), were designed with the PyroMark Assay Design software (Qiagen, Hilden, Germany). Primers and sequences to analyze are listed in Table 2. *FOXP3* is located on the X-chromosome (Xp11.23), resulting in hemizygosity in male subjects and random inactivation of the second X-chromosome in females, subsequently methylation data was stratified according to sex. Bisulfite conversion of DNA was performed using the EpiTect 96 Bisulfite Kit (Qiagen, Hilden, Germany) according to the manufacturer’s protocol. PCR amplifications were performed on a Tetrad 2 cycler (BioRad, Munich, Germany) with an initial denaturation step at 95°C for 5 min, 40 cycles of 95°C for 30 s, primer-specific annealing temperature of 60°C for 30 s, 72°C for 45 s, and a final extension step at 72°C for 10 min. The reaction mixture consisted of 2.5 μl 10x PCR buffer with MgCl_2_, 0.5 μl 10 mM dNTP mix, 1.25 μl of each forward and reverse primer (final concentration 0,5 μM), 0.2 μl (final concentration 1 U) Taq DNA polymerase (Roche Diagnostics, Mannheim, Germany), 18.3 μl PCR-grade water, and 1 μl template DNA (75 ng). PCR products were visualized by electrophoresis on 1.5% agarose gel.

**Table 2:** Primers used for bisulfite pyrosequencing.

Gene	Primer	Sequence (5’-3’)	Number of CpGs
***FOXP3***	forward	Biotin-AGTTTGGTTTGTGGGAAATTGTT	
	reverse	ACCCTATTATCTCATTAATACCTCTCA	
	Sequence 1	ATAAAAACAAAATTATTTTTAATA	1
	Sequence 2	AAATTATTAAAAAAAAAAAATCTAC	4
***FOXP3* Enhancer**	forward	ATGAAGGGGAGGAGGAAG	
	reverse	Biotin-CCTCCAACTCCACCATAAC	
	Sequence 1	GAGGAAGAGGAGGTT	4
	Sequence 2	GGGTTTTATTTGGTTTTTATATT	7

Bisulfite pyrosequencing was performed on a PyroMarkTMQ96 MD Pyrosequencing System with the PyroMark Gold Q96 CDT Reagent Kit (Qiagen, Hilden, Germany). Our experimental methodology relied on simultaneous treatment of control and study samples in order to avoid batch effect and technical variability which is estimated around 1–2% of this assay. Bisulfite conversion, PCR amplifications and pyrosequencing of control and study samples were performed together. The sequences analyzed by bisulfite pyrosequencing are listed in Table 3. Data analysis was done with the Pyro Q-CpG software (Qiagen, Hilden, Germany).

**Table 3:** Sequences analyzed by bisulfite pyrosequencing.

Gene	Sequence analyzed*
***FOXP3* CpG number Sequence 1**	CRTAACAATTTCCCACAAACCAAACTA
	.1
***FOXP3* CpG number Sequence 2**	RACTTCCACACCRTACAACRTAATTTTTCTTCTCRATATA
	1. . . .. . .. . ..2. . .. . .3. . . .. . . .. . .. . .4
***FOXP3* Enhancer CpG number Sequence 1**	TGTTTYGAGTTTTTATYGTTGTGTTTYGTTTTYGT
	. . . ..1. . . .. . .. . .2. . . .. . . ..3. . . ..4
***FOXP3* Enhancer CpG number Sequence 2**	TTYGTTGTYGTTYGYGTYGGGTYGTTTGGAGYGT
	..1. . . ..2. . .3.4..5. . . .6. . . .. . . .7

*analyzed CpG sites are underlined and consecutively numbered

### Statistical analysis

Shapiro-Wilk test was used to test for normal distribution, before applying Student’s t-test for normally-distributed and non-parametric Mann-Whitney-U for not normally-distributed independent variables. Correlations between variables were identified by Spearman's rank correlation coefficient. A generalized linear multivariate regression model was generated by stepwise regression to infer the influence of gender, depression, medication with antidepressants and smoking, adjusted for the chronological age (age at blood withdrawal) of the patient. Step-down exclusion was performed by excluding variables with the standardized regression coefficient closest to 0 until a significant regression model was generated. Disease duration was excluded from the regression model due to strong correlation with age (Table 4). A p-value ≤0.05 was considered statistically significant. Given the explorative nature of the study, no correction for multiple testing was performed. All statistical analyses were performed with SPSS Version 23.0 (Chicago, IL, USA).

**Table 4:** Methylation of *FOXP3* Promoter and Enhancer regions.

	Females		p-value^1^	Males		p-value^2^
	Patients	Controls		Patients	Controls	
**Promoter sequence 1**	69.06 ± 4.94	65.54 ± 9.19	0.008**	68.32 ± 5.39	68.30 ± 4.60	0.899
Position CpG 1	69.06 ± 4.94	65.54 ± 9.19	0.008**	68.32 ± 5.39	68.30 ± 4.60	0.899
**Promoter sequence 2 (all 4 CpGs)**	79.09 ± 2.12	78.37 ± 1.84	0.022*	70.61 ± 3.47	70.52 ± 3.08	0.802
Position CpG 1	90.21 ± 2.12	90.20 ± 1.86	0.621	82.04 ± 3.41	82.28 ± 2.90	0.856
Position CpG 2	75.76 ± 2.36	74.89 ± 1.89	0.054	68.81 ± 3.31	68.74 ± 3.58	0.941
Position CpG 3	75.05 ± 3.88	74.13 ± 2.98	0.011**	69.13 ± 3.57	68.73 ± 3.25	0.675
Position CpG 4	75.01 ± 3.31	74.27 ± 2.65	0.140	62.67 ± 5.50	62.34 ± 3.88	0.676
**Promoter sequences 1+2 (all 5 CpGs)**	77.01 ± 2.40	75.80 ± 2.56	0.005**	70.16 ± 3.77	70.08 ± 3.24	0.761
**Enhancer sequence 1 (all 4 CpGs)**	39.69 ± 3.25	39.81 ± 2.21	0.246	2.09 ± 0.91	1.93 ± 0.59	0.356
Position CpG 1	40.03 ± 2.85	40.42 ± 3.41	0.376	2.00 ± 1.59	2.05 ± 2.04	0.997
Position CpG 2	37.48 ± 3.18	38.38 ± 2.85	0.291	2.68 ± 0.81	2.58 ± 0.83	0.316
Position CpG 3	40.89 ± 3.79	42.09 ± 2.71	0.202	1.87 ± 0.39	1.85 ± 0.54	0.838
Position CpG 4	36.36 ± 8.99	37.93 ± 6.12	0.636	1.79 ± 1.42	1.28 ± 0.63	0.598
**Enhancer sequence 2 (all 7 CpGs)**	38.00 ± 2.68	38.18 ± 1.77	0.749	1.31 ± 0.44	1.37 ± 0.69	0.899
Position CpG 1	37.74 ± 4.05	37.62 ± 3.31	0.652	1.79 ± 1.38	2.38 ± 2.58	0.517
Position CpG 2	41.00 ± 3.39	40.18 ± 3.56	0.364	2.53 ± 1.18	2.73 ± 1.47	0.707
Position CpG 3	37.42 ± 3.59	38.71 ± 3.22	0.093	0.94 ± 0.78	0.84 ± 0.62	0.624
Position CpG 4	30.56 ± 4.61	30.65 ± 3.31	0.699	0.67 ± 0.57	0.71 ± 0.49	0.440
Position CpG 5	44.51 ± 3.25	44.80 ± 2.63	0.988	0.79 ± 0.94	0.68 ± 0.75	0.366
Position CpG 6	40.19 ± 3.46	40.70 ± 2.47	0.792	0.67 ± 0.41	0.77 ± 0.92	0.943
Position CpG 7	34.66 ± 4.02	34.57 ± 3.06	0.823	1.81 ± 0.96	1.52 ± 0.70	0.188
**Enhancer sequences 1+2 (all 11 CpGs)**	38.26 ± 2.48	38.73 ± 1.64	0.577	1.60 ± 0.46	1.59 ± 0.55	0.825

Values are given in mean percentages methylation ± standard deviation. For all positions higher methylation was found in females compared to males in patients (p<0.0001) and controls (p<0.0001) except for CpGs in promoter sequence 1 in position 1 (female patients versus male patients: p = 0.401; female controls versus male controls: p = 0.159).

^1^Comparison between female patients and female controls

* = significant at p≤0.05

** = significant at p≤0.01.

^2^Comparison between male patients and male controls: not significant.

## Results

### Lower TREC counts in panic disorder patients

**Fig 2:**
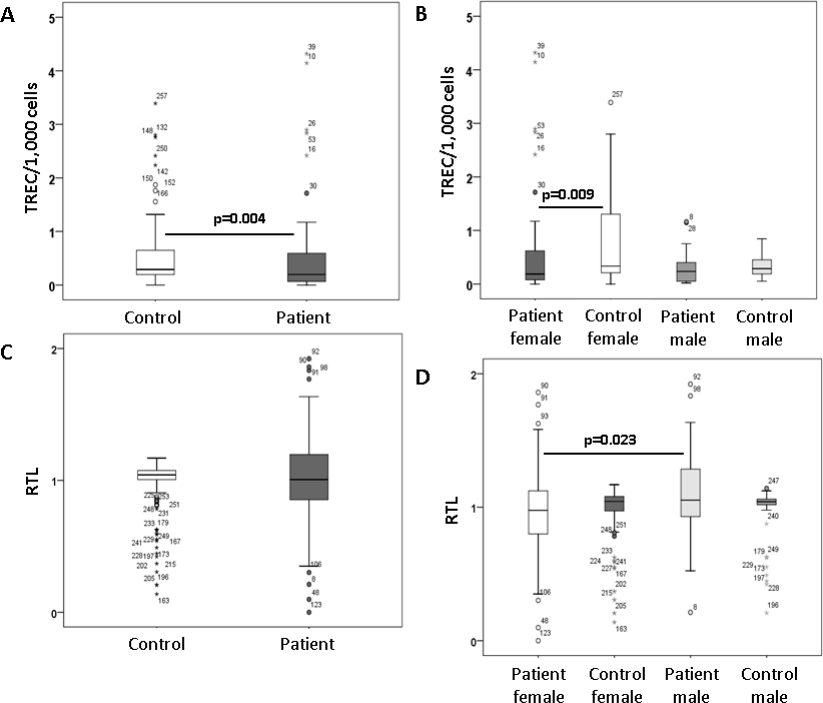
T cell receptor excision circles (TRECs). T cell receptor excision circles (TRECs) per 1,000 peripheral blood mononuclear cells (PBMCs) were significantly lower including all patients compared to controls (A) and in female patients with panic disorder compared to healthy female controls (B) (Mann-Whitney U test). Relative telomere lengths (RTLs) are shown in patients and compared to controls (C) and in female patients with panic disorder compared to healthy female controls (D) (Mann-Whitney U test). Circles (outliers) and asterisks (extreme values) represent individuals coded with numbers.

Patients (mean 0.49 ± 0.81) showed significantly lower TRECs/1,000 cells (mean 0.99 ± 1.90) compared to healthy controls (HC) (p = 0.004), which was due to significantly lower TRECs in female patients (Fig 2). In patients, TRECs negatively correlated with age (R = -0.245; p = 0.025). An association of lower TREC counts with age was shown for both, female (R = -0.378; p = 0.019) and male patients (R = -0.578; p = 0.024). Regression analysis (R^2^ = 0.051, p = 0.045) including age (p = 0.086) and gender (p = 0.468) revealed that having panic disorder (p = 0.016) was an independent factor for lower TRECs. Slightly lower TRECs were seen in patients treated with antidepressants compared to those without any medication (p = 0.024). Performing regression analysis (R^2^ = 0.109, p = 0.019) in patients only, age (p = 0.031) was an independent factor for lower TRECs, whereas gender (p = 0.162), smoking (p = 0.727), and comorbid depression (p = 0.925) or antidepressants (p = 0.483) had no significant association.

### *FOXP3* promoter and enhancer methylation

**Fig 3:**
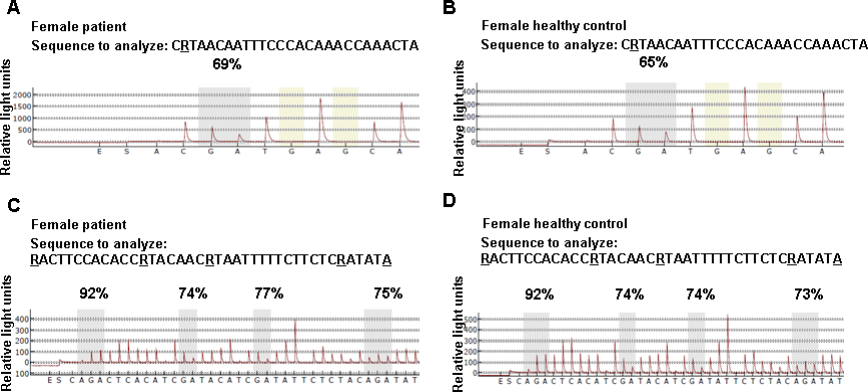
Methylations at specific CpG sites in a representative female patient and a healthy female control person. Methylations at one specific CpG site in *FOXP3* promoter sequence 1 (position 1), and at four independent CpG sites in *FOXP3* promoter sequence 2 (positions 1 to 4) of a representative female patient (A, C) and a healthy female control person (B, D) were quantified in a single pyrosequencing run. Position-specific information in the context of the analyzed sequence presents broad-sequence methylation patterns (% methylation). The built-in quality control sites (highlighted in yellow) consisting of cytosines converted to thymines demonstrate full bisulfite conversion of the treated DNA.

Significantly higher *FOXP3* promoter methylation across all five CpGs as well as particularly at sequence 1, CpG 1, and sequence 2, CpGs 2 and 3 was discerned in female patients compared to HC (Table 4, exemplarily shown in Fig 3). Methylation at sequence 2, CpGs 2 and 3 highly correlated with each other (R = 0.698; p = 0.0001), but not with sequence 1, CpG 1. Regression analysis (R^2^ = 0.059; p = 0.060) revealed that having panic disorder was an independent factor for higher *FOXP3* methylation across all five CpGs (p = 0.020) in female patients. Lower methylation across all five CpGs was seen in patients on antidepressants (mean methylation 75.94 ± 2.63%) compared to those without any medication (77.78 ± 1.98%) (p = 0.014), with high significance at *FOXP3* promoter sequence 2, CpG 4 (antidepressants: 73.58 ± 2.39%; no antidepressants: 76.06 ± 3.66%; p = 0.004). Using a regression model for methylation across all five CpGs (R^2^ = 0.276; p = 0.024) in female patients, antidepressants (p = 0.004) and age (p = 0.027) (depression p = 0.921; smoking p = 0.273) showed the strongest associations, with lower *FOXP3* promoter methylation correlating with antidepressant medication and higher age. No significant difference between female patients and female HC could be demonstrated for *FOXP3* enhancer methylation (Table 4). Categorization of female patients into smokers, into patients with depression and into patients on antidepressants did not reveal any differences in *FOXP3* enhancer methylation.

In males, *FOXP3* promoter and enhancer methylation were not different between patients and HC (Table 4). Smoking male patients had significantly higher methylation at *FOXP3* enhancer, sequence 2, CpG 5 (mean methylation 0.98 ± 0.73%) compared to non-smoking male patients (0.78 ± 1.19%) (p = 0.030). In male patients, significantly higher methylation was determined at *FOXP3* enhancer, sequence 2, CpG 4 (0.84 ± 0.70%) and 5 (1.16 ± 1.33%) in the case of depression compared to male patients without depression (position 4 (0.51 ± 0.37%) and 5 (0.51 ± 0.33%); p = 0.039 and p = 0.006, respectively). Similarly, higher methylation was seen at *FOXP3* enhancer, sequence 2, CpG 5 in male patients on antidepressants (1.15 ± 1.35%) compared to those without antidepressants (0.53 ± 0.31%) (p = 0.013). Methylation at *FOXP3* enhancer, sequence 2, CpG 4 highly correlated with methylation at *FOXP3* enhancer, sequence 2, position 5 (R = 0.675; p = 0.0001). Multiple regression analysis (R² = 0.345; p = 0.032) showed that methylation at *FOXP3* enhancer, sequence 2, CpG 5 was weakly influenced by smoking (p = 0.067) and older age (p = 0.073) but not by depression (p = 0.267) or antidepressants (p = 0.868).

### Relative telomere lengths

Relative telomere lengths (RTLs) were not different between patients and HC. However, within the patient group, smokers had significantly shorter telomeres (0.91 ± 0.30) compared to non-smokers (1.07 ± 0.37) (p = 0.018) and females (0.96 ± 0.34) had shorter telomeres than males (1.10 ± 0.32) (p = 0.017), although age and distribution of females were not significantly different between smokers and non-smokers. Stratifying for female patients aged ≥35 years, difference in RTLs was significant between smokers (0.84 ± 0.32) and non-smokers (1.12 ± 0.34) (p = 0.010). In patients, regression analysis (R² = 0.150; p = 0.008) identified smoking (p = 0.004), female gender (p = 0.011) and age (p = 0.041) as factors associated with shorter telomeres, whereas depression (p = 0.106) and antidepressants (p = 0.446) were less important. Interestingly, hypermethylation at *FOXP3* promoter, sequence 2, CpGs 2 (R = -0.495; p = 0.0001) and 3 (R = -0.309; p = 0.035) correlated with shorter telomeres in female patients.

## Discussion

The present study demonstrated significant lower TRECs in both female and male panic disorder patients as well as significant hypermethylation of the *FOXP3* promoter region in female patients with panic disorder as compared to healthy controls. No difference in relative telomere length was discerned between patients and controls, but significantly shorter telomeres in females, smokers and individuals aged ≥35 years of the patient group.

Whereas diminished thymic function and dysfunction of immune regulation by FoxP3+ Tregs is well recognized in stress-induced depression in mice [40,48], less is known in human anxiety and particularly panic disorder. Lower TRECs—as presently observed in patients with panic disorder—indicate a reduced thymic output of recent thymic emigrants, which may account for increased infection rates and inflammatory disorders in patients with panic disorder as known from clinical observation [12,49,50]. Our results underline the role of an impaired thymic function and/or highly proliferating peripheral T cells contributing to the lower TREC numbers and the strong influence of age on TRECs as seen in the patient group. As TRECs are not only a marker for thymic function but also for peripheral replication with dilution of TRECs, relative telomere lengths were evaluated to estimate replicative activity of peripheral lymphocytes [20]. Although no association of telomere lengths were identified with the categorical diagnosis of panic disorder, telomere lengths were shorter in females and smokers within the panic disorder sample and associated with higher age. In contrast to T cell-specific TRECs, telomeres were measured in samples from total leukocytes and may be greatly influenced by other subpopulations than naive T cells. This may also allow speculation for an effect of smoking habits, steroids or female hormones or other environmental factors for confounding the telomere results [45].

Diminished thymic function has been associated with inflammatory diseases [13,14,17] due to compensatory proliferative mechanisms in the periphery and increased Th1 and Th17 responses in relation to a dysfunctional Treg activity. Additionally, lower output of thymic-dependent naturally occurring Tregs may result in lower suppression of inflammatory responses in the periphery. Our study revealed hypermethylation of the *FOXP3* promotor region—potentially resulting in reduced immunosuppressive Treg function—in female but not in male patients with panic disorders, corroborates the idea of a prematurely aged immune system in this particular patient subgroup. Although a strong sex bias of autoimmunity, with most autoimmune diseases predominantly affecting females, is well known [51,52], the underlying mechanisms are not well understood. The absence of a second (inactivated) X chromosome in males, sex hormones, and sex-specific differences in gene regulation due to internal and external (i.e. environmental) factors, all can influence the susceptibility to disease. Particularly, hormone factors may explain the higher differences regarding methylation status of CpG regions within the *FOXP3* promoter [53,54] and lower TRECs in females [55].

Interestingly, besides older age, antidepressants were found to be associated with a relative demethylation at specific CpGs within the *FOXP3* promoter region. An association between age and *FOXP3* hypomethylation with increased immunosuppressive Treg function has been suggested by a recent study in mice [56]. Likewise, *FOXP3* demethylation could constitute a molecular correlate of beneficial effects of antidepressants on the immune system in panic disorder. This notion is supported by first therapy-epigenetic studies showing dynamic methylation changes after successful antidepressant or even psychotherapeutic treatment in depression or anxiety disorders [57–59]. However, it has to be noted that the CpGs observed to be relatively demethylated in association with antidepressants were different from CpGs associated with panic disorder in the present sample. In males, methylation was higher at specific CpGs in the *FOXP3* enhancer region in smokers as well as in patients with comorbid depression compared to non-smoking patients or patients without depression, respectively. Antidepressants were associated with higher methylation at the same CpG position. However, given only minor *FOXP3* enhancer methylation in general (see Table 4) in male subjects and an underpowered sample size of the male subsample, these results are to be considered with caution.

In addition to the present cross-sectional study design, other factors influencing inflammation, such as fat tissue mass in obese patients, comorbid diabetes or latent infections (e. g. Cytomegalovirus infections) contributing to an immune-risk-phenotype [13,60], may limit the interpretation of our results. Also, it is unclear whether the findings from this study may be interpreted as primary or secondary events to panic disorder, although a stress-induced, and thus secondary neuroendocrinological effect of panic disorder on the immune system particularly in older females, may be a reasonable explanation for reduced thymic activity and hypermethylation of *FOXP3*. This interpretation is also supported by mouse models [61–64] and observations in humans [49]. Although the expression level of *FOXP3* could not be analyzed because of limitations in available samples many groups showed that demethylation of promoter and enhancer regions of *FOXP3* corresponds to expression of FoxP3 protein in peripheral lymphocytes [28,65]. The biological relevance of 5’ upstream enhancer was demonstrated by the ability of methotrexate treatment to restore defective Treg function through demethylation in rheumatoid arthritis patients [65]. Differences in the methylation of *FOXP3* promotor are quite small just above the background noise of the method and may be unable to explain all alterations found in patients with panic disorder. However, at least small differences in promoter methylation may influence the accessibility of the gene and, thus, the ability to provide stable *FOXP3* expression as shown by others [65]. Although the transactivation activity of FOXP3 promoter appears to be weak, a weak transactivation activity may help prevent promiscuous FOXP3 induction [66].

One limitation of our study is that lymphocytes were not separated into CD25highCD4+ T cells, defining mainly nTregs, and in naive and other T cell subpopulations, as a differentiated methylation pattern at the *FOXP3* enhancer region was found on activated and only transiently FoxP3-expressing T cells with impermanent change of methylation status [34]. This may explain that we were not able to find significant differences in the methylation of CpG regions of the analyzed enhancer regions and only small differences in the promoter region as several T cell subpopulations with different methylation levels at the *FOXP3* promotor regions may contribute to the methylation results. Another important limitation to mention is the fact that bisulfite sequencing cannot discriminate between 5-methylcytosine and 5-hydroxymethylcytosine. Therefore, the output from bisulfite sequencing cannot solely be interpreted as showing only 5-methylcytosines, but it could also include the 5-hydroxymethylcytosines. 5-hydroxymethylcytosine has been postulated to play an important role in the process of demethylation [67], where 5-hydroxymethylcytosine facilitates passive demethylation and in turn promotes gene transcription.

Regarding the RTL data, it has to be taken into account that several factors could influence the outcome such as current inflammatory state as measurable by C-reactive protein levels, anti-inflammatory medications, paternal age, menopause, exercise, diet or childhood trauma. However, unfortunately, these data were not available and thus, have not been corrected for in the present study. Mechanisms and directions of interaction between anxiety and immune function as well as their relation to an increased risk of mortality and morbidity, also considering early as well as recent life events need to be further evaluated in longitudinal studies.

In summary, the present study noted reduced TRECs in panic disorder patients compared to controls as well as *FOXP3* hypermethylation in patients with panic disorder potentially reflecting impaired thymus and immunosuppressive Treg function. From the present results, we expect that female and older patients with panic disorder may show particularly strong effects regarding immunosenescence and its role in development of age-associated diseases, such as cardiovascular events, autoimmune disorders, cancer and infectious rates accounting for the known increased morbidity and mortality of anxiety disorders. Targeted prevention and early treatment of anxiety disorders could therefore aid in mitigating their detrimental effects on the immune system and thereby in lowering the risk of diseases associated with age such as coronary heart disease, cardiovascular death and cancer.
